# A Calorie-Restricted Ketogenic Diet Reduces Cerebral Cortex Vascularization in Prepubertal Rats

**DOI:** 10.3390/nu11112681

**Published:** 2019-11-05

**Authors:** Andrea Viggiano, Rosaria Meccariello, Antonietta Santoro, Carmine Secondulfo, Francesca Felicia Operto, Marcellino Monda, Giangennaro Coppola

**Affiliations:** 1 Department of Medicine, Surgery and Dentistry “Scuola Medica Salernitana”, University of Salerno, 84081 Baronissi, Italy; ansantoro@unisa.it (A.S.); csecondulfo.md@gmail.com (C.S.); gcoppola@unisa.it (G.C.); 2 Department of Movement Sciences and Wellbeing, University of Naples “Parthenope”, 80133 Naples, Italy; meccariello@uniparthenope.it; 3 Clinic of Child and Adolescent Neuropsychiatry, S. Giovanni di Dio and Ruggi d’Aragona Hospital, University of Salerno, 84131 Salerno, Italy; opertofrancesca@gmail.com; 4 Department of Experimental Medicine, Section of Human Physiology, University of Campania “Luigi Vanvitelli”, 80138 Naples, Italy; marcellino.monda@unicampania.it

**Keywords:** ketogenic diets, epilepsy, growth, angiogenesis, cerebral cortex, substantia nigra

## Abstract

The antiepileptic effect of ketogenic diets is acknowledged but its mechanism of action is poorly understood. The present work aimed to evaluate possible effects of a calorie-restricted ketogenic diet (CRKD) on brain growth and angiogenesis in normal prepubertal rats. Two groups of prepubertal rats were fed with a standard diet (group 1) or a CRKD (group 2) for ten weeks. Then, rats were sacrificed and the thickness for the following structures was evaluated by histology: (1) cerebral cortex, (2) deep cerebral white matter, and (3) substantia nigra. The capillary density was also evaluated within: (1) cerebral cortex, (2) dentate gyrus of the hippocampus, (3) periaqueductal grey matter, and (4) substantia nigra. The results showed a smaller thickness of all the areas examined and a reduced capillary density within the cerebral cortex in the CRKD-treated group compared to the control group. These findings suggest an association between reduced angiogenesis within the cerebral cortex and the antiepileptic effects of CRKD.

## 1. Introduction

Ketogenic diets (KDs) are well known for their efficacy in the treatment of several kinds of epilepsies. In particular, a greater efficacy has been reported for epileptic encephalopathies [1,2,3] rather than for partial seizures [4]. The mechanism of action of KDs is poorly understood [5]. Ketone bodies have been investigated for possible antiepileptic effects [6,7], but it has also been described that KDs are effective when they are calorie-restricted and administered to young subjects [8].Thus, calorie restriction appears to have an important role in the antiepileptic mechanism [9,10]. Calorie restriction can obviously reduce body weight gain at prepubertal ages; in fact, growth retardation due to calorie-restricted KDs has been reported in animal models [7,10,11] and in humans [12,13,14]. Thus, it can be questioned whether a calorie-restricted KD (CRKD) could produce an antiepileptic effect due to a reduction in the growth of some structures within the brain. The rationale for such question is further supported by at least two evidence: (1) CRKD can reduce the growth of a brain glioma due to a reduction in angiogenesis [15,16]; (2) the inhibition of angiogenesis can prevent the development of epilepsy in a pilocarpine rat model [17].

The present experiment aimed to evaluate the growth of forebrain structures and microvasculature after a period of CRKD.

## 2. Materials and Methods

### 2.1. Animals

Two groups of seven male Wistar rats (Harlan Laboratories, Udine, Italy), aging 5 weeks, weighting 108 ± 13 g (mean ± S.D.), were used. They were housed in standard temperature and humidity conditions with a 12 h light–dark cycle (lights on from 07:00 to 19:00). Animals were fed with standard food ad libitum (control group) or with a CRKD (CRKD-treated group) as previously described [18]. Briefly, the CRKD was a food mix composed of more than 78% by fat (Bio-Serv cF3666, Frenchtown, NJ, USA) and was limited to approximately 90% of the calculated daily calorierequirement. Rats were fed with the above diets for ten weeks, then they were sacrificed by anesthetic overdose. All procedures fulfilled the requirements of the European Communities Council Directive of 22 September 2010 (2010/63EU). The research was approved by the local ethical committee of the University of Salerno and by the Ministry of Health of the Italian Government.

### 2.2. Histology

After sacrifice, rats ware perfused with 4% formaldehyde/phosphate buffered saline (PBS). Brains were immediately removed and stored in fixative, then equilibrated with 30% sucrose/PBS for at least one week. Coronal sections (20 μm in thickness) were obtained with a cryostat at the level of the midbrain (6 mm posterior to bregma) and mounted on microscope slides (Super Frost Ultra Plus, Menzel, Germany). From each animal, twenty sections were taken and alternatively processed for myelin staining or for microvasculature marking by immunofluorescence.

**Figure 1:**
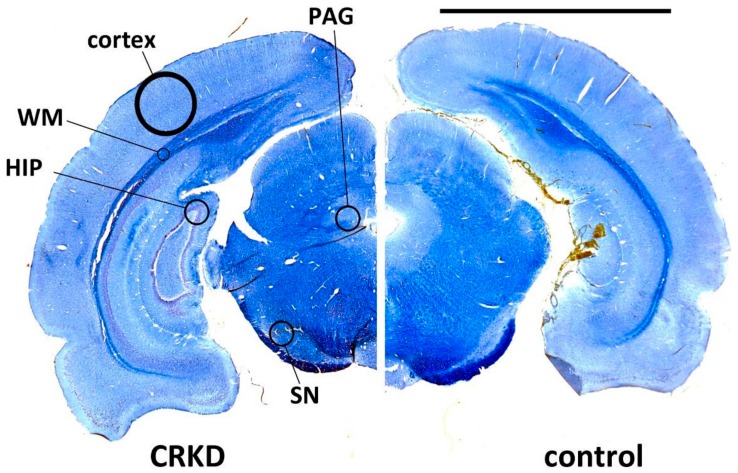
Coronal sections from a calorie-restricted ketogenic diet (CRKD)-treated rat brain and a control rat brain stained with Eriochrome. The circles show the sites for measuring the dimensions of cerebral cortex (cortex), deep cerebral white matter (WM), and substantia nigra (SN) and the sites for capillary density evaluation by immunofluorescence within the dental gyrus of the hippocampus (HIP), the cerebral cortex (cortex), the periaqueductal gray matter (PAG), and the SN. Bar = 5 mm.

For myelin staining, sections were first dehydrated by immersion in ethanol 70% (2 min), 90% (2 min), and 100% (4 min), then left in xylene for 60 min. The sections were then rehydrated by immersion in ethanol 100% (4 min), 90% (2 min), 70% (2 min), and water (2 min). This treatment was necessary for Erichrome staining of myelin. Myelin staining was obtained by immersion in an Eriochrome solution (20 min), consisting of Eriochrome Cyanine R (CI 43,820, Sigma-Aldrich, Milan, Italy) 0.1 g/100 mL + FeCl_3_ 0.224 g/100 mL + sulfuric acid 0.5 mL/100 mL. Subsequent steps were: washing with water; immersion in a fresh FeCl_3_ solution (FeCl_3_-6H_2_O 4 g/100 mL, 4 min); washing with water; immersion in a Neutral Red solution (Neutral Red 1 g/100 mL adjusted to pH 5.19 with NaOH, 10 min); washing with water; immersion in ethanol 70% (2 min), 90% (2 min), and 100% (2 min) and in xylene; coverslip mounting. These sections were used to measure the thickness of: (1) cerebral cortex (primary visual cortex), (2) deep cerebral white matter, (3) substantia nigra (SN; Figure 1).

For microvasculature immunofluorescence, sections were first processed for antigen retrieval by immersion in sodium citrate buffer 10 mM pH 6.0 + Tween20 0.05% at 95 °C for 40 min. Subsequent steps were: washing with PBS; incubation with normal donkey serum 10% (Jackson, West Grove, PA, USA) + TritonX100 0.1% for 1 h; washing with PBS; overnight incubation with mouse anti-GLUT1 antibody 1:50 (ab40084, Abcam, Cambridge, UK); washing with PBS; incubation with Alexa Fluor 647-donkey anti-mouse antibody for 1 h (Jackson, West Grove, PA, USA); washing with PBS; coverslip mounting. Digital images were acquired through a fluorescent microscope for subsequent evaluation of the capillary density within: (1) cerebral cortex (primary visual cortex), (2) dentate gyrus of the hippocampus (HIP), (3) periaqueductal grey matter (PAG), (4) SN. Capillary density was esteemed by: (1) total area covered by capillaries per microscopic field; (2) total number of capillaries per microscopic field; (3) mean dimension of capillaries, esteemed, for each microscopic field, by the ratio between the total capillary area and the number of capillaries; (4) mean capillary branching ratio, given, for each microscopic field, by the ratio between the total number of branching points and the number of capillaries.

### 2.3. Statistical Analysis

For each animal and for each histological measure, the mean value of the measures taken from 10 sections was considered. Data are expressed as means ± S.E. Statistically significant differences between the two experimental groups were evaluated by the Student’s *t*-test.

## 3. Results

### 3.1. Body Weight and Brain Dimensions

As expected, the CRKD produced a significant impairment of growth. The mean body weights after 10 weeks of diet were 369 ± 21 g for the control group and 156 ± 7 g for the CRKD-treated group; the Student’s *t*-test demonstrated a significant difference for the mean body weight between the two groups (*p* < 0.01).

**Figure 2:**
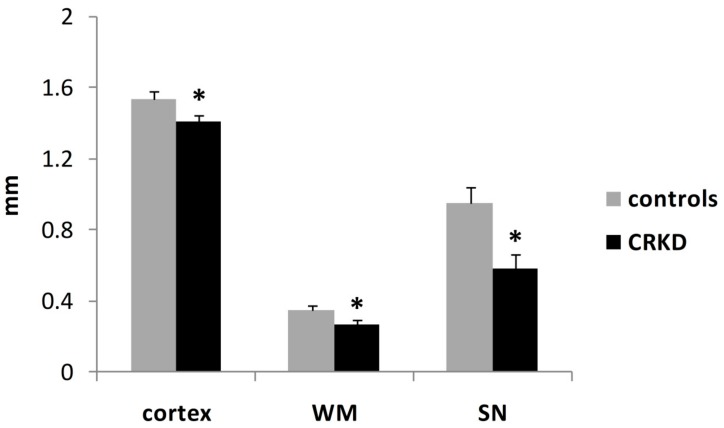
Mean thickness of cerebral cortex (primary visual cortex), deep cerebral white matter (WM), substantia nigra (SN). * *p* < 0.05, compared to the control group.

Compared to controls, the CRKD-treated group showed thinner cerebral cortex, deep cerebral white matter and substantia nigra (Figure 2). The Student’s *t*-test demonstrated a significant difference in the mean thickness of all these structures between the two groups (*p* < 0.05).

### 3.2. Capillary Density

**Figure 3:**
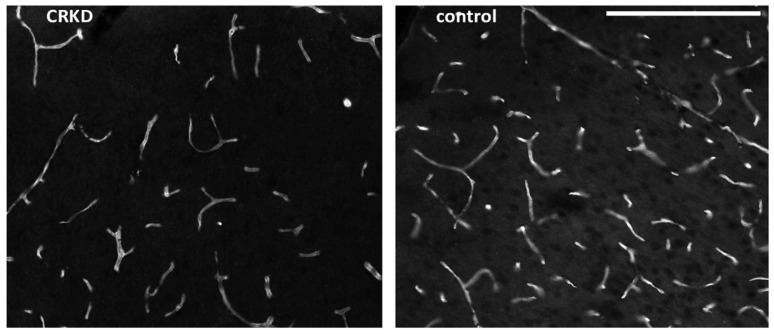
Images of the cerebral cortex of a CRKD-treated rat and from a control rat. The sections were marked with anti-GLUT1 resulting in a revelation of all microvessels. Bar = 0.2 mm.

An example of immunofluorescence image of capillaries is shown in Figure 3.

**Figure 4:**
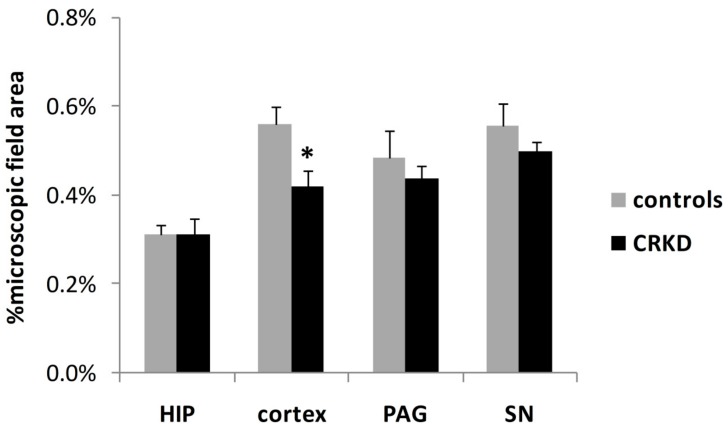
Mean total capillary area (percentage of microscopic field area) within cerebral cortex (primary visual cortex), dentate gyrus of the hippocampus (HIP), periaqueductal grey matter (PAG) and substantia nigra (SN). * *p* < 0.05, compared to the control group.

The total capillary area per microscopic field was smaller in the cerebral cortex of CRKD-treated rats, but not in the other areas examined (Figure 4). The Student’s *t*-test demonstrated a significant difference in the mean capillary area of the cerebral cortex between the two groups (*p* < 0.05).

**Figure 5:**
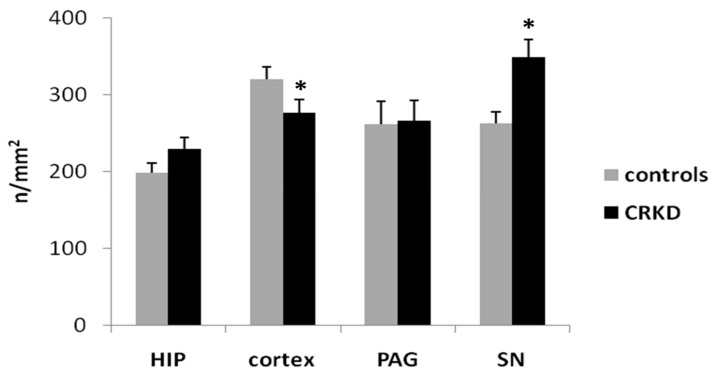
Mean capillary density within cerebral cortex (primary visual cortex), dentate gyrus of the hippocampus (HIP), periaqueductal grey matter (PAG), and substantia nigra (SN). The capillary density was obtained by counting the number of capillaries per microscopic field and dividing it by the microscopic field area. * *p* < 0.05, compared to the control group.

The number of capillaries per microscopic field in the cerebral cortex of the CRKD-treated group was smaller than in the control group. Conversely, the number of capillaries in the SN was greater in the CRKDgroup (Figure 5); the number of capillaries in the HIP and the PAG did not differ between the two groups. The Student’s *t*-test demonstrated a significant difference between the two groups for the number of capillaries within the cerebral cortex and the SN (*p* < 0.05).

**Figure 6:**
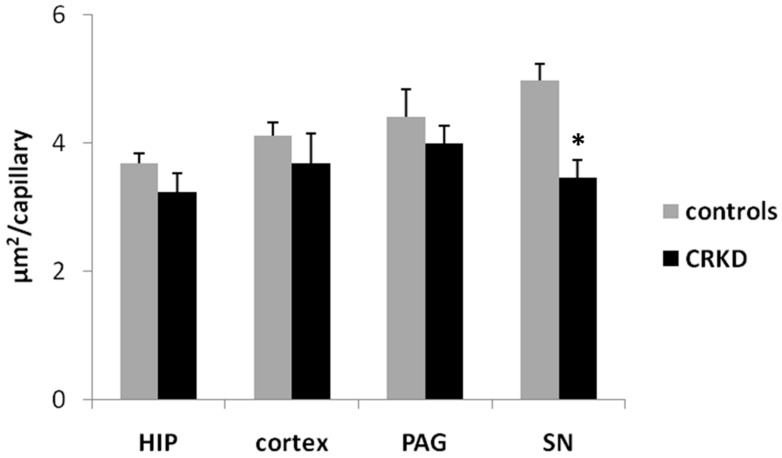
Mean single capillary area within cerebral cortex (primary visual cortex), dentate gyrus of the hippocampus (HIP), periaqueductal grey matter (PAG), and substantia nigra (SN). The mean single capillary dimension was obtained for each microscopic field by the ratio between the total capillary area and the number of capillaries. * *p* < 0.01, compared to the control group.

The mean capillary dimension was smaller in the CRKDgroup compared to the control group within the SN but did not differ within the other brain structures examined (Figure 6). The Student’s *t*-test demonstrated a significant difference between the two groups for the dimension of capillaries within the SN (*p* < 0.01).

**Figure 7:**
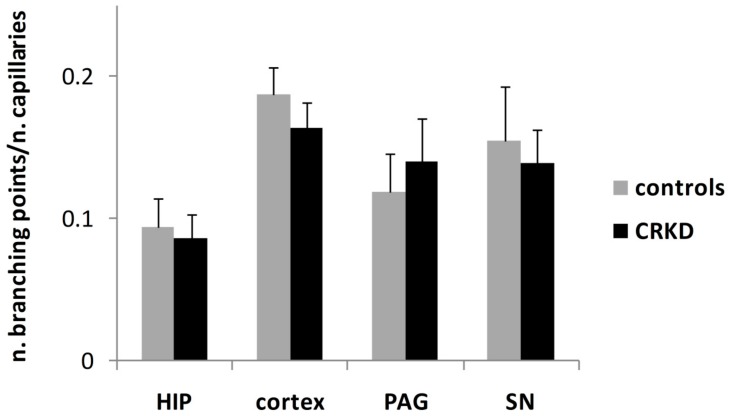
Mean branching ratio of capillaries within cerebral cortex (primary visual cortex), dentate gyrus of the hippocampus (HIP), periaqueductal grey matter (PAG), and substantia nigra (SN). No significant differences were seen between the two groups for any brain area.

The mean capillary branching ratio did not differ between the two experimental groups in any of the brain structures examined (Figure 7).

## 4. Discussion

The results of the present study demonstrated that the CRKD produced a significant reduction in brain growth involving both white matter and grey matter (Figure 2). To our knowledge, this is the first report of the effects of a CRKD on the dimensions of brain structures. This finding agrees with previous studies reporting a growth-limiting effect of CRKD in animal models [7,10,11] and in humans [12,13,14].

It has also been demonstrated that CRKD can affect capillary density, but this effect appears to affect some specific brain areas. In particular, it has been found that CRKD can reduce capillary density within the cerebral cortex. In fact, both the number and the area covered by capillaries were reduced in CRKD-treated rats (Figure 4 and Figure 5). This finding agrees with previous studies reporting a reduction of angiogenesis within brain gliomas after a CRKD treatment [15,16]. Calorie restriction is probably a key factor for the reduction of angiogenesis in all these models. In fact, in other experiments using a ketogenic diet fed ad libitum it was observed an increase in vascular brain density; it can also be noted that in this case body weight was not affected by the diet [19].

CRKD seems also to affect the vasculature within the SN, but in this structure it has been found an increase in the number of capillaries (Figure 5) without any significant change in the total area covered by capillaries (Figure 4), meaning that capillaries within the SN were smaller on average (Figure 6). This apparent discrepancy could eventually be ascribed to the timing of the treatment used in the present model. In fact, it has been shown that at birth angiogenesis is still ongoing within some forebrain structures, including the cerebral cortex, but not in all brain areas [20], so it can be hypothesized that angiogenesis within the substantia nigra takes place in an early stage of development, prior to the time of the treatment of the present model. Unfortunately, to our knowledge, there is a lack of data about the timing of angiogenesis during development within the substantia nigra, so further studies are needed to verify this point.

At present, it is not possible to describe the mechanism by which CRKD causes a reduction of angiogenesis, but it is very likely that the key points can be (1) the limited amount of amino acids supplied, which will obviously limit the growth of any kind of tissue, and (2) the reduced production of insulin due to the low level of blood glucose. In fact, reduced levels of insulin and insulin growth factor 1 (IGF-1) due to CRKD have been confirmed in humans [21,22,23]. Moreover, the involvement of insulin in brain angiogenesis is supported by literature and this mechanism is even supposed to play a role in the pathogenesis of Alzheimer’s disease [24]. Another possible mechanism of action of CRKD on brain angiogenesis could be through a reduction of IGF-1, which has been proven to be necessary for remodeling of brain vessels [25].

Another open question to clarify is the reason for the selective effects of CRKD on the vascularity of cerebral cortex and SN. Part of this finding could eventually be ascribed to the method used in the present study, which could not be sensible enough to reveal effects on the angiogenesis of other brain areas. It is evident, in any case, that the effects on cerebral cortex and SN were the strongest. This finding partly agrees with the results of a paper by Zeller et al. who reported that “hippocampal CA1 area shows a different development without any significant increase in relative capillary density during maturation” [26]; thus, it can be argued that CRKD can reduce the rate of angiogenesis within those brain regions in which angiogenesis is normally stimulated during maturation, like in cerebral cortex [20]. Another experiment demonstrated that the pharmacological inhibition of angiogenesis blocked the increase in vascularity within the hippocampus and prevented epilepsy after a pilocarpine injection [17]; thus, the possibility that the reduction in angiogenesis due to CRKD can have an antiepileptic effect deserve much attention in future studies.

The prevalence of the anti-angiogenic effect on the cerebral cortex and on the SN resembles the clinical evidence that CRKD has greater efficacy in epileptic encephalopathies [1,2,3] and in patients with neuronal migration disorders [27], rather than in partial seizures [4]. In fact, epileptic encephalopathies and neuronal migration disorders arise from generalized dysfunctions of the cerebral cortex and/or of structures involved in the spreading of seizures, namely the SN. The role of the SN in spreading of seizures is acknowledged [28,29,30,31], and involvement of the SN in the effects of CRKD has been demonstrated in previous experiments [11,32].

## 5. Conclusions

In conclusion, the present study demonstrated a reduction of body weight and a reduction of the dimensions of brain structures in juvenile rats treated with CRKD for 4 weeks. This finding deserves further investigations to understand the mechanisms and whether these are involved in the antiepileptic effect of ketogenic diets.
